# Sleep Duration and the Risk of Fatty Liver Disease: A Systematic Review and Meta-analysis

**DOI:** 10.1038/srep31956

**Published:** 2016-08-23

**Authors:** Na Shen, Peng Wang, Weiming Yan

**Affiliations:** 1 Department of Laboratory Medicine, Tongji Hospital, Tongji Medical College, Huazhong University of Science and Technology, Wuhan 430030, China; 2 Institute and Department of Infectious Disease, Tongji Hospital, Tongji Medical College, Huazhong University of Science and Technology, Wuhan, China

## Abstract

Recent studies have reported inconsistent results on the association between sleep duration and the risk of fatty liver disease (FLD). Thus, we quantitatively evaluated this association by performing a systematic review and meta-analysis, based on a comprehensive electronic search in databases of PubMed, Web of Science, EMBASE, ClinicalTrials.gov, Wanfangdata and Chinese National Knowledge Infrastructure (CNKI) (updated to April 2016). Multivariate adjusted odds ratios (ORs) and 95% confidence intervals (95% CIs) were extracted and pooled by using a random-effects model. Eight eligible studies involving 97,371 participants were included. We found that neither short nor long sleep duration was significantly related with FLD risk. For short sleep duration, the pooled OR was 1.17 (95% CI = 0.98–1.38), and for long sleep duration, the pooled OR was 1.01 (95% CI = 0.72–1.41). Subgroup analyses by sex, outcome, and exposure reference also did not identify any effect of sleep duration on FLD onset. In summary, our findings suggested that short or long sleep duration was not significantly associated with FLD risk. Further cohort studies with refined designs are still warranted to validate our results.

Fatty liver disease (FLD) is a pathological condition in which excessive triglycerides accumulate in liver cells due to many different causes. In various countries, FLD prevalence ranges from 10% to 24% in general population^1^. Nowadays, FLD, including non-alcoholic fatty liver disease (NAFLD), is becoming the most common chronic liver disease worldwide, resulting in an enormous clinical and economic burden for public health^2^,^3^. Many efforts have been made to investigate FLD pathogenesis. Although mechanisms are still unclear, many lifestyle factors, including weight gain^4^, television viewing^5^ and sleep disorder^6^, are reportedly related to FLD risk.

Sleep patterns have changed with societal development, which may influence quality of life. Recent studies have suggested a link between sleep duration and long-term health status, including adult depression^7^, type 2 diabetes^8^ and cardiovascular outcomes^9^. However, the association between sleep duration and FLD risk was largely inconsistent. Kim *et al*. found that women with short sleep duration more frequently suffered from NAFLD^10^. Miyake *et al*. reported that less sleep was associated with reduced NAFLD risk in men^11^. However, Hsieh *et al*. observed an insignificant association between short sleep duration and FLD risk in men^12^, and Imaizumi *et al*. observed similar results in men and women^13^. Long sleep duration was reportedly protective against FLD^12^, but this result was not supported by subsequent research^13^.

Insufficient power in individual studies may lead to these conflicting results. Thus, we systematically reviewed relevant literature and performed this meta-analysis to investigate the association of short and long sleep durations with FLD risk.

## Results

### Literature screening

**Figure 1:**
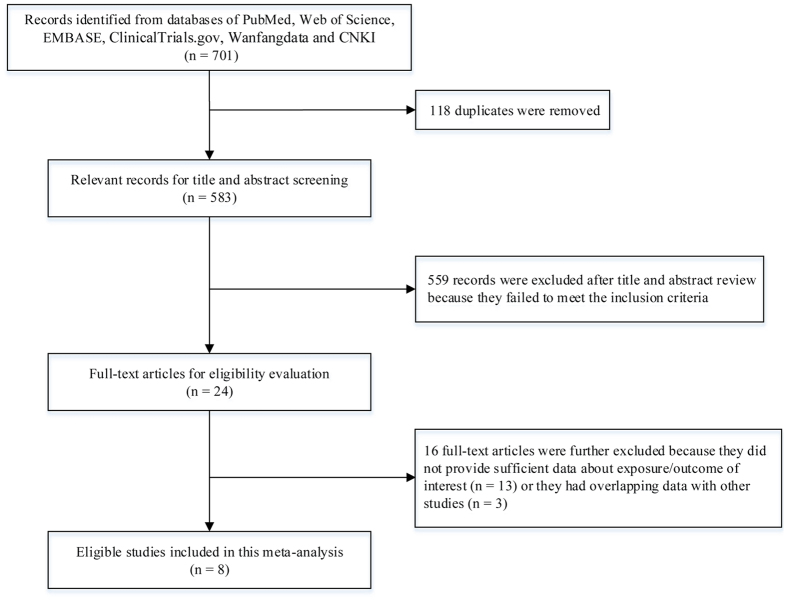
A flowchart of literature search and study selection. Abbreviations: CNKI, Chinese national knowledge infrastructure.

As shown in Fig. 1, 701 relevant records were identified from databases of PubMed, Web of Science, EMBASE, ClinicalTrials.gov, Wanfangdata and Chinese National Knowledge Infrastructure (CNKI). After removing 118 duplicated records, 583 records underwent title and abstract screening. Then 559 records were excluded because they were experimental studies, review articles, comments, or irrelevant to sleep duration and outcome of interest. The remaining 24 studies were evaluated in detail for eligibility by full-text review. Of them, 16 studies were further excluded due to providing insufficient data about exposure/outcome of interest or reporting overlapping data with other studies. Finally, we included eight eligible studies in this meta-analysis^10^,^11^,^12^,^13^,^14^,^15^,^16^,^17^.

### Characteristics of included studies

The characteristics of the included studies are presented in Table 1. All eligible studies were based on health check-up cohorts, six of which were conducted in Asia^10^,^11^,^12^,^13^,^14^,^16^ and two of which were conducted in Europe^17^ or America^15^. In total, 97,371 participants with a mean age range from 21.7 to 62.4 years were involved in our meta-analysis. Information on sleep duration was collected by self-administered questionnaire. The primary outcomes were fatty liver and NAFLD, which were determined by ultrasonography or liver enzymes tests. ORs and 95% CIs extracted from individual studies were adjusted by age and other covariates. Four studies^10^,^11^,^13^,^14^ reported results separately by sex and one^16^ reported by obstructive sleep apnea (OSA) status. Thus, we treated them as independent datasets.

### Sleep duration and FLD risk

**Figure 2:**
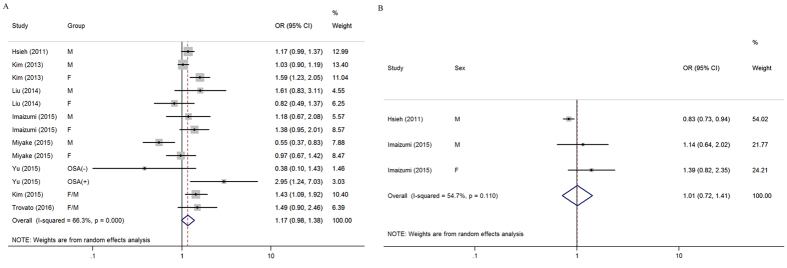
Overall forest plots. (**A**) Forest plot of the association between short sleep duration and FLD risk. (**B**) Forest plot of the association between long sleep duration and FLD risk. Abbreviations: M, male; F, female; OSA, obstructive sleep apnea.

As a modifiable factor, sleep duration usually exhibits a U-shaped relationship with illness^7^,^8^,^9^. Thus, we explored the effects of short and long sleep duration on the risk of FLD. By combining 13 independent datasets from the eight studies^10^,^11^,^12^,^13^,^14^,^15^,^16^,^17^ (involving 97,371 subjects), we identified that participants with the shortest sleep duration did not have significant risk for FLD, compared with those who slept longer (the pooled OR = 1.17, 95% CI = 0.98–1.38). Study heterogeneity was significant with high level (*P*_*heterogeneity*_ < 0.001, *I*^*2*^ = 66.3%) (Fig. 2A). We also investigated the effect of long sleep duration on FLD risk. By combining three independent datasets from the two studies^12^,^13^ (including 10,329 subjects), we showed that compared to normal sleep duration (5~7 hours), long sleep duration did not increase or decrease the risk of FLD (the pooled OR = 1.01, 95% CI = 0.72–1.41). The heterogeneity among studies was moderate (*P*_*heterogeneity*_ = 0.110, *I*^*2*^ = 54.7%) (Fig. 2B).

### Meta-regression and subgroup analyses

Given the significant and high heterogeneity observed when pooling data about short sleep duration, we performed univariate meta-regression analysis to explore potential sources (Table 2) and made the scatter-plots to illustrate the relationships between short sleep duration and each potential moderator (Supplemental Figure S1). Publication year, mean age, sex, exposure reference (5~8 hours or not) and region (Asian or not) were considered influencing factors, but the results did not identify any significant factor (all *P* values > 0.05). We further investigated potential effect modification for short sleep duration according to sex, outcome and reference exposure. As shown in Table 3, less sleep, whether in males or females, did not modify FLD risk (Male: the pooled OR = 1.02, 95% CI = 0.81–1.29; Female: the pooled OR = 1.20, 95% CI = 0.89–1.62). Because seven of eight included studies focused on NAFLD^10^,^11^,^13^,^14^,^15^,^16^,^17^, we examined the effect of short sleep duration on NAFLD risk and found no significant relationship (the pooled OR = 1.17, 95% CI = 0.95–1.43). In the reference exposure subgroup, we focused on studies that considered normal sleep duration (5~8 hours) as a reference, and also failed to observe any significant association (the pooled OR = 1.01, 95% CI = 0.76–1.35). Subgroup analysis was not conducted for long sleep duration because of the limited number of independent datasets. In addition, quality scores of Newcastle-Ottawa assessment indicated that all included studies were of high quality (all quality scores ≥ 5).

### Sensitivity analyses and publication bias

**Figure 3:**
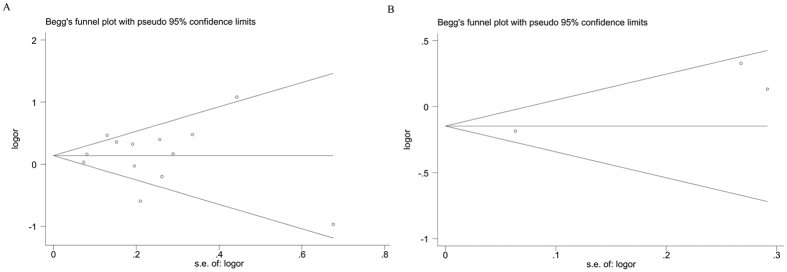
Funnel plots. (**A**) Funnel plot of the association between short sleep duration and FLD risk. (**B**) Funnel plot of the association between long sleep duration and FLD risk.

Results for short or long sleep duration were not appreciably altered based on one-way sensitivity analyses (Supplemental Table S1). Although results changed somewhat by removing the male dataset from Miyake *et al*.^11^, pooled ORs stratified by sex were still not significant, with low or moderate heterogeneity. That suggested that the results in this meta-analysis were relatively robust. For assessments of publication bias, we did not observe any asymmetry in funnel plots (Fig. 3A,B). Moreover, Begg’s and Egger’s tests did not provide any evidence of publication bias (For short sleep duration: *P*_*Egger’s test*_ = 0.870, *P*_*Begg’s test*_ = 0.951. For long sleep duration: *P*_*Egger’s test*_ = 0.189, *P*_*Begg’s test*_ = 0.602).

## Discussion

In this present meta-analysis, we demonstrated that neither short nor long sleep duration was significantly associated with FLD risk. Further subgroup analyses showed that this lack of association was not appreciably modified by sex, outcome, or reference exposure.

Many studies on sleep duration and FLD risk focus on its association with obesity, a well-known pathogenic factor for FLD onset. A previous meta-analysis suggested that short sleep duration was related to obesity, whereas long sleep duration had no effect^18^. However, recent studies have shown that poor sleep quality may also play an important role in obesity^19^,^20^,^21^. Moreover, Kim *et al*. found a significant relationship between poor sleep quality and NAFLD in both men and women^10^. In addition, sleep disorders, including obstructive sleep apnoea (OSA) have been revealed as risk factors for FLD^6^,^22^. To date, several studies have investigated the effects of sleep duration on FLD risk^10^,^11^,^12^,^13^,^14^,^15^,^16^,^17^, but many of them were not fully adjusted for confounders in design or analysis. This may explain why we found no association between sleep duration and FLD risk.

A major strength of our meta-analysis was its large overall sample size, including 97,371 participants. Besides, pooled data were extracted from multivariate adjusted models in original studies, which reduced confounding bias to some extent. In addition, our findings were strengthened by accordant results from subgroup analyses. These advantages made our pooled estimate more exact and credible.

However, several limitations should also be acknowledged in our meta-analysis. Firstly, the reference exposure of sleep duration were inconsistent among the included studies, which contributed to the significant heterogeneity during data integration. Therefore, we applied a random-effects model in all analyses in this meta-analysis. Secondly, the included studies measured sleep duration based on a self-reported questionnaire rather than actigraphy monitoring. Although a good correlation was shown between the two measurements^23^, it is difficult to avoid non-robust estimations of the association between sleep duration and FLD risk because of subjective bias. Thirdly, the included studies were observational designs in which exposure misclassification possibly influenced the pooled results. Additionally, based on the different adjusted factors among studies, our pooled results may be biased by residual or unmeasured confounders in the original studies. Finally, only two studies comprising three datasets were included the evaluation of an association between long sleep duration and FLD risk. Although the pooled results were stable according to sensitivity analysis and there was no evidence of publication bias, the results should be treated with caution, and further studies are needed to confirm them.

In summary, our meta-analysis suggested that there was no association between short or long sleep duration and FLD risk. Further subgroup analyses supported this conclusion. However, more cohort studies with large-scale and refined designs are still warranted to further validate our results.

## Methods

### Literature search and study selection

This current meta-analysis was performed following the guidelines of the Preferred Reporting Items for Systematic Reviews and Meta-Analyses (PRISMA) statement^24^. We systematically searched the literature in databases of PubMed, Web of Science, EMBASE, ClinicalTrials.gov, Wanfangdata and Chinese National Knowledge Infrastructure (CNKI) through April 2016 to identify studies that examine the association between sleep duration and FLD risk, without any restriction. The search strategy included items of “sleep” and “fatty liver”, and it is described in Supplemental Table S2. To retrieve extra citations, we also manually checked the reference lists from relevant articles and reviews.

Studies were considered eligible if they (1) were cross-sectional, retrospective, or prospective designs and (2) reported odds ratios (ORs) and 95% confidence intervals (CIs) for the effects of sleep duration on FLD or sufficient data to extrapolate risk estimates. For studies involving data that overlapped with other studies, we selected the studies with newer results or larger sample sizes. Two authors independently conducted the literature searches and study selection, and any discrepancy was resolved by consensus.

### Data extraction and quality assessment

Two independent authors extracted the following items from each eligible study: (1) general information, including first author’s name, publication year, and country; (2) study characteristics, including age, sex, and numbers of participants; (3) reference exposure and exposure of interest; (4) outcomes and outcome assessment; (5) covariates adjusted in the individual study; and (6) risk measures of association, including ORs and 95% CIs. Discrepancies were resolved by group discussion. We also evaluated the quality of each study using the Newcastle-Ottawa scale^25^ (Supplemental Table S3).

### Statistical Analysis

Multivariate adjusted ORs and 95% CIs were recorded from each eligible study to estimate the association between sleep duration and FLD risk. These ORs were pooled with a random-effects model based on the inverse of variance method^26^. We used the Cochran’s Q test to examine between-study heterogeneity, which was also quantified by the *I*^*2*^ index^27^. Heterogeneity is considered significant if *P* < 0.10, and it is defined as low, moderate or high based on *I*^*2*^ values of 25%, 50% and 75%, respectively^28^. Meta-regression analysis was used to investigate potential sources of heterogeneity. To further explore potential effects of sleep duration on FLD risk, we conducted subgroup analyses and stratified data using modifiers that included sex, outcomes, and reference exposure. Publication bias was assessed by Egger’s regression test^29^ and Begg’s rank correlation test^30^. In addition, we performed sensitivity analyses to evaluate the robustness of the pooled results. Specifically, the pooled OR was estimated again by removing one included study at a time to check stability. All these analyses above were performed by using Stata 12.1 software (College Station, TX, USA), with a two-sided P ≤ 0.05 as the significance level unless otherwise specified.

## Additional Information

**How to cite this article**: Shen, N. *et al*. Sleep Duration and the Risk of Fatty Liver Disease: A Systematic Review and Meta-analysis. *Sci. Rep.* **6**, 31956; doi: 10.1038/srep31956 (2016).

## Supplementary Material

Supplementary Information

## Figures and Tables

**Table 1:** Characteristics of included studies in this meta-analysis.

Study	Country	Sex, male (%)	Participants	Mean age (years)	Reference exposure	Exposure of interest	Outcome	Outcome assessment	Adjusted variables	Newcastle-Ottawa score
Hsieh *et al*.^12^	Japan	M, 100	8157	51.6	5~<7 hours	<5 hours; ≥7 hours	Fatty liver	ultrasonography	age, poor sleep (defined as difficulty of getting to sleep or awakening easily)	5
Kim *et al*.^10^	Korea	F/M, 57.6	45293	39.7	>7 hours	≤5 hours	NAFLD	ultrasonography	age, smoking, alcohol intake, physical activity, systolic blood pressure, education level, marital status, presence of job, sleep apnea, loud snoring, and BMI	7
Liu *et al*.^14^	China	F/M, 39.8	20746	62.4	>8 hours	≤5 hours	NAFLD	ultrasonography	age, smoking, alcohol intake and BMI	7
Imaizumi *et al*.^13^	Japan	F/M, 33.7	2172	60.9	6 ~≤7 hours	≤6 hours; >8 hours	NAFLD	ultrasonography	age, smoking, no breakfast, snacking, regular exercise and BMI	6
Miyake *et al*.^11^	Japan	F/M, 27.5	2429	40.4	7~8 hours	≤6 hours	NAFLD	ultrasonography	age, BMI, SBP, TG, HDL-c, FPG, UA, ALT, Cre, snacking habit, and periodic exercise habit	6
Yu *et al*.^16^	Korea	F/M, 57.2	621	56.6	≥5 hours	<5 hours	NAFLD	LAI value < 5 HU	age, sex, exercise, alcohol, smoking, DM, HTN, CVD, and BMI	7
Kim *et al*.^15^	United States	F/M, 48.7	17245	46.0	≥9 hours	≤5 hours	NAFLD	ultrasonography	age, gender, ethnicity, BMI, waist circumference, educational level, marital status, economic status, smoking, diabetes and hypertension and sleep quality	6
Trovato *et al*.^17^	Italy	F/M, 35.3	708	21.7	≥25^th^ lower percentile sleep hours	<25^th^ lower percentile sleep hours	NAFLD	ultrasonography	BMI, waist circumference, oversized clothes, sedentary lifestyle, cigarette smoking, coffee, daily frequency of eating	7

Abbreviations: M, male; F, female; LAI, liver attenuation index; BMI, body mass index; SBP, systolic blood pressure; TG, triglycerides; HDL-c, high-density lipoprotein cholesterol; FPG, fasting plasma glucose; UA, uric acid; ALT, alanine aminotransferase; Cre, creatinine; DM, diabetes mellitus; HTN, hypertension; CVD, cardiovascular disease.

**Table 2:** Univariate meta-regression analysis of the association between short sleep duration and FLD risk.

Factors^a^	Coefficient	Standard error	*P*
Publication year	−0.007	0.081	0.934
Mean age	0.001	0.010	0.921
Sex	−0.160	0.225	0.500
Reference exposure	0.244	0.211	0.271
Region	−0.264	0.287	0.379

^a^Reference exposure referred to 5~8 hours or not; Region referred to Asian or non-Asian area.

**Table 3:** Subgroup analyses of the association between short sleep duration and FLD risk.

	n^a^	OR (95% CI)	*P*_*heterogeneity*_	*I*^*2*^ (%)
Sex				
Male	5	1.02 (0.81–1.29)	0.011	69.3
Female	4	1.20 (0.89–1.62)	0.050	61.7
Outcome				
NAFLD	12	1.17 (0.95–1.43)	<0.001	69.0
Reference exposure				
5~8 hours	5	1.01 (0.76–1.35)	0.010	69.7

Abbreviation: NAFLD, non-alcoholic fatty liver disease.

^a^n referred to individual datasets.

## References

[b1] Angulo P. Nonalcoholic fatty liver disease. The New England journal of medicine 346, 1221–1231, 10.1056/NEJMra011775 (2002). 11961152

[b2] Lazo M. & Clark J. M. The Epidemiology of Nonalcoholic Fatty Liver Disease: A Global Perspective. Seminars in Liver Disease 28, 339–350, 10.1055/s-0028-1091978 (2008). 18956290

[b3] Younossi Z. M. . Global Epidemiology of Non-Alcoholic Fatty Liver Disease-Meta-Analytic Assessment of Prevalence, Incidence and Outcomes. Hepatology, 10.1002/hep.28431 (2015). 26707365

[b4] Katrina Loomis A. . Body mass index and risk of non-alcoholic fatty liver disease: Two electronic health record prospective studies. The Journal of clinical endocrinology and metabolism, jc20153444, 10.1210/jc.2015-3444 (2015). PMC4803162 26672639

[b5] Helajarvi H. . Television viewing and fatty liver in early midlife. The Cardiovascular Risk in Young Finns Study. Annals of medicine 47, 519–526, 10.3109/07853890.2015.1077989 (2015). 26362414

[b6] Sookoian S. & Pirola C. J. Obstructive Sleep Apnea Is Associated with Fatty Liver and Abnormal Liver Enzymes: a Meta-analysis. Obesity Surgery 23, 1815–1825, 10.1007/s11695-013-0981-4 (2013). 23740153

[b7] Zhai L., Zhang H. & Zhang D. Sleep duration and depression among adults: a meta-analysis of prospective studies. Depression and anxiety 32, 664–670, 10.1002/da.22386 (2015). 26047492

[b8] Shan Z. . Sleep duration and risk of type 2 diabetes: a meta-analysis of prospective studies. Diabetes Care 38, 529–537, 10.2337/dc14-2073 (2015). 25715415

[b9] Cappuccio F. P., Cooper D., D’Elia L., Strazzullo P. & Miller M. A. Sleep duration predicts cardiovascular outcomes: a systematic review and meta-analysis of prospective studies. European heart journal 32, 1484–1492, 10.1093/eurheartj/ehr007 (2011). 21300732

[b10] Kim C. W. . Sleep duration and quality in relation to non-alcoholic fatty liver disease in middle-aged workers and their spouses. Journal of hepatology 59, 351–357, 10.1016/j.jhep.2013.03.035 (2013). 23578884

[b11] Miyake T. . Short sleep duration reduces the risk of nonalcoholic fatty liver disease onset in men: a community-based longitudinal cohort study. Journal of gastroenterology 50, 583–589, 10.1007/s00535-014-0989-0 (2015). 25120172

[b12] Hsieh S. D., Muto T., Murase T., Tsuji H. & Arase Y. Association of short sleep duration with obesity, diabetes, fatty liver and behavioral factors in Japanese men. Internal medicine (Tokyo, Japan) 50, 2499–2502 (2011). 10.2169/internalmedicine.50.5844 22041348

[b13] Imaizumi H. . The Association between Sleep Duration and Non-Alcoholic Fatty Liver Disease among Japanese Men and Women. Obesity facts 8, 234–242, 10.1159/000436997 (2015). 26138724 PMC5644852

[b14] Liu L. The association between sleep duration and non-alcoholic fatty liver disease: a cross-sectional study, Huazhong University of Science & Technology, (2014).

[b15] Kim D. . Short sleep duration is associated with nonalcoholic fatty liver disease in US adults. Hepatology 62, 1258A (2015).

[b16] Yu J. H. . Obstructive sleep apnea with excessive daytime sleepiness is associated with non-alcoholic fatty liver disease regardless of visceral fat. The Korean journal of internal medicine 30, 846–855, 10.3904/kjim.2015.30.6.846 (2015). 26552460 PMC4642014

[b17] Trovato F. M. . Fatty liver disease and lifestyle in youngsters: diet, food intake frequency, exercise, sleep shortage and fashion. Liver international: official journal of the International Association for the Study of the Liver 36, 427–433, 10.1111/liv.12957 (2016). 26346413

[b18] Wu Y., Zhai L. & Zhang D. Sleep duration and obesity among adults: a meta-analysis of prospective studies. Sleep Med 15, 1456–1462, 10.1016/j.sleep.2014.07.018 (2014). 25450058

[b19] Kim M. Association between objectively measured sleep quality and obesity in community-dwelling adults aged 80 years or older: a cross-sectional study. Journal of Korean medical science 30, 199–206, 10.3346/jkms.2015.30.2.199 (2015). 25653493 PMC4310948

[b20] McNeil J. . Objectively-measured sleep and its association with adiposity and physical activity in a sample of Canadian children. Journal of sleep research 24, 131–139, 10.1111/jsr.12241 (2015). 25266575

[b21] Rahe C., Czira M. E., Teismann H. & Berger K. Associations between poor sleep quality and different measures of obesity. Sleep Med 16, 1225–1228, 10.1016/j.sleep.2015.05.023 (2015). 26429750

[b22] Musso G. . Association of obstructive sleep apnoea with the presence and severity of non-alcoholic fatty liver disease. A systematic review and meta-analysis. Obesity reviews: an official journal of the International Association for the Study of Obesity 14, 417–431, 10.1111/obr.12020 (2013). 23387384

[b23] Spiegel K., Knutson K., Leproult R., Tasali E. & Van Cauter E. Sleep loss: a novel risk factor for insulin resistance and Type 2 diabetes. Journal of applied physiology (Bethesda, Md.: 1985) 99, 2008–2019, 10.1152/japplphysiol.00660.2005 (2005). 16227462

[b24] Moher D., Liberati A., Tetzlaff J. & Altman D. G. Preferred reporting items for systematic reviews and meta-analyses: the PRISMA Statement. Open medicine: a peer-reviewed, independent, open-access journal 3, e123–e130 (2009). PMC3090117 21603045

[b25] Stang A. Critical evaluation of the Newcastle-Ottawa scale for the assessment of the quality of nonrandomized studies in meta-analyses. Eur J Epidemiol 25, 603–605, 10.1007/s10654-010-9491-z (2010). 20652370

[b26] Harris R. J. . metan: fixed- and random-effects meta-analysis. Stata Journal 8, 3–28 (2008).

[b27] Higgins J. P. & Thompson S. G. Quantifying heterogeneity in a meta-analysis. Statistics in medicine 21, 1539–1558, 10.1002/sim.1186 (2002). 12111919

[b28] Higgins J. P., Thompson S. G., Deeks J. J. & Altman D. G. Measuring inconsistency in meta-analyses. BMJ (Clinical research ed.) 327, 557–560, 10.1136/bmj.327.7414.557 (2003). PMC192859 12958120

[b29] Egger M., Davey Smith G., Schneider M. & Minder C. Bias in meta-analysis detected by a simple, graphical test. BMJ (Clinical research ed.) 315, 629–634 (1997). 10.1136/bmj.315.7109.629 PMC2127453 9310563

[b30] Begg C. B. & Mazumdar M. Operating characteristics of a rank correlation test for publication bias. Biometrics 50, 1088–1101 (1994). 7786990

