# Supplementary material for: Sleep Duration and the Risk of Fatty Liver Disease: A Systematic Review and Meta-analysis
Source: Sci Rep. 2016 Aug 23;6:31956. doi: 10.1038/srep31956 (PMC4994071; doi:10.1038/srep31956)

Sleep Duration and the Risk of Fatty Liver Disease: A Systematic Review and Meta-analysis

Na Shen[1*], Peng Wang[2*], Weiming Yan[2]

[1] Department of Laboratory Medicine, Tongji Hospital, Tongji Medical College, Huazhong University of Science and Technology, Wuhan 430030, China.

[2] Institute and Department of Infectious Disease, Tongji Hospital, Tongji Medical College, Huazhong University of Science and Technology, Wuhan, China.

Correspondence to:

Na Shen, Department of Laboratory Medicine, Tongji Hospital, Tongji Medical College, Huazhong University of Science and Technology, Wuhan 430030, China. Tel. : +86-27-83663414, E-mail: shenna@tjh.tjmu.edu.cn

[*] These authors contributed equally to this work.

Supplemental Table S1. Sensitivity analyses of the association between sleep duration and FLD risk

Study removed	OR (95%CI)	$P_{heterogeneity}$	I^2 (%)
Short sleep duration			
Hsieh (2011)-M	1.17 (0.95-1.43)	< 0.001	69.0
Kim (2013)-M	1.19 (0.97-1.45)	0.001	65.8
Kim (2013)-F	1.12 (0.94-1.33)	0.003	61.6
Liu (2014)-M	1.15 (0.96-1.37)	<0.001	68.2
Liu (2014)-F	1.19 (1.00-1.42)	<0.001	67.6
Imaizumi (2015)-M	1.17 (0.97-1.39)	<0.001	69.1
Imaizumi (2015)-F	1.15 (0.96-1.38)	<0.001	68.2
Miyake (2015)-M	1.24 (1.07-1.43)	0.018	52.0
Miyake (2015)-F	1.19 (0.99-1.42)	<0.001	68.4
Yu (2015)-OSA(-)	1.18 (1.00-1.40)	0.001	66.6
Yu (2015)-OSA(+)	1.13 (0.96-1.34)	0.001	64.5
Kim (2015)	1.14 (0.95-1.37)	<0.001	66.8
Trovato (2016)	1.15 (0.96-1.37)	<0.001	68.2
Long sleep duration			
Hsieh (2011)-M	1.27 (0.86-1.87)	0.618	0.0
Imaizumi (2015)-M	1.01 (0.62-1.64)	0.062	71.3
Imaizumi (2015)-F	0.86 (0.71-1.03)	0.290	10.5

Abbreviations: M, male; F, female; OSA, obstructive sleep apnea.

Supplemental Table S2. Search strategy

Database: PubMed

1. ("sleep"[MeSH Terms] OR "sleep"[All Fields]) AND duration[All Fields]
2. ("sleep"[MeSH Terms] OR "sleep"[All Fields]) AND ("time"[MeSH Terms] OR "time"[All Fields])
3. "disorders of excessive somnolence"[MeSH Terms] OR ("disorders"[All Fields] AND "excessive"[All Fields] AND "somnolence"[All Fields]) OR "disorders of excessive somnolence"[All Fields] OR "hypersomnia"[All Fields]
4. "sleep initiation and maintenance disorders"[MeSH Terms] OR ("sleep"[All Fields] AND "initiation"[All Fields] AND "maintenance"[All Fields] AND "disorders"[All Fields]) OR "sleep initiation and maintenance disorders"[All Fields] OR "insomnia"[All Fields]
5. 1 OR 2 OR 3 OR 4
6. "fatty liver"[MeSH Terms] OR ("fatty"[All Fields] AND "liver"[All Fields]) OR "fatty liver"[All Fields]
7. 5 AND 6

Database: Web of Science

1. TOPIC: (sleep duration), Timespan=All years, Search language=Auto
2. TOPIC: (sleep time), Timespan=All years, Search language=Auto
3. TOPIC: (hypersomnia) , Timespan=All years, Search language=Auto
4. TOPIC: (insomnia), Timespan=All years, Search language=Auto
5. 4 OR 3 OR 2 OR 1
6. TOPIC: (fatty liver) , Timespan=All years, Search language=Auto
7. 6 AND 5

Database: EMBASE

1. fatty AND ('liver'/exp OR liver)
2. steatohepatitis
3. 1 OR 2
4. 'sleep duration' OR 'sleep time'

5. iInsomnia
6. hypersomnia
7. 4 OR 5 OR 6
8. 3 AND 7

Database: ClinicalTrials.gov

fatty liver AND (sleep OR hypersomnia OR insomnia)

Database: Wanfangdata

1. 睡眠时间 + 失眠 + 过度睡眠 * Date:-2016
2. "脂肪肝" * Date:-2016
3. 1 AND 2

Database: Chinese National Knowledge Infrastructure (CNKI)

1. 检索条件：((主题=中英文扩展(睡眠时间) 或者 主题=中英文扩展(失眠)) 或者 主题=中英文扩展(过度睡眠)) (模糊匹配),专辑导航：全部; 数据库：文献 跨库检索
2. 检索条件：(主题=中英文扩展(脂肪肝)) (精确匹配),专辑导航：全部; 数据库：文献 跨库检索
3. 1 AND 2

Supplemental Table S3. Quality assessment of included studies by Newcastle-Ottawa scale

Study	Selection (0-4)				Comparability (0-2)		Exposure (0-3)			Score
	ADC	RC	SC	DC	SCB	AF	AE	SMA	NRE	
Hsieh et al. (2011) [12]	1	1	1			1		1		5
Kim et al. (2013) [10]	1	1	1	1	1	1		1		7
Liu et al. (2014) [14]	1	1	1	1	1	1		1		7
Imaizumi et al. (2015) [13]	1	1	1		1	1		1		6
Miyake et al. (2015)[11]	1	1	1		1	1		1		6
Yu et al. (2015) [16]	1	1	1	1	1	1		1		7
Kim et al. (2015) [15]	1	1	1		1	1		1		6
Trovato et al. (2016) [17]	1	1	1	1	1	1		1		7

Abbreviations: ADC, adequate case definition; RC, Representativeness of the cases; SC, Selection of Controls; DC, Definition of Controls; SCB, study controls for body mass index (BMI); AF, study controls for any additional factors (age, sex, smoking, etc.); AE, Ascertainment of exposure; SMA, Same method of ascertainment for cases and controls; NRE, Non-Response rate.

Supplemental Figure S1. Meta-regression plots for potential moderators on short sleep duration. **(A)** Meta-regression plots for publication year on short sleep duration. **(B)** Meta-regression plots for mean age on short sleep duration. **(C)** Meta-regression plots for sex on short sleep duration. **(D)** Meta-regression plots for reference exposure on short sleep duration. **(E)** Meta-regression plots for region on short sleep duration.

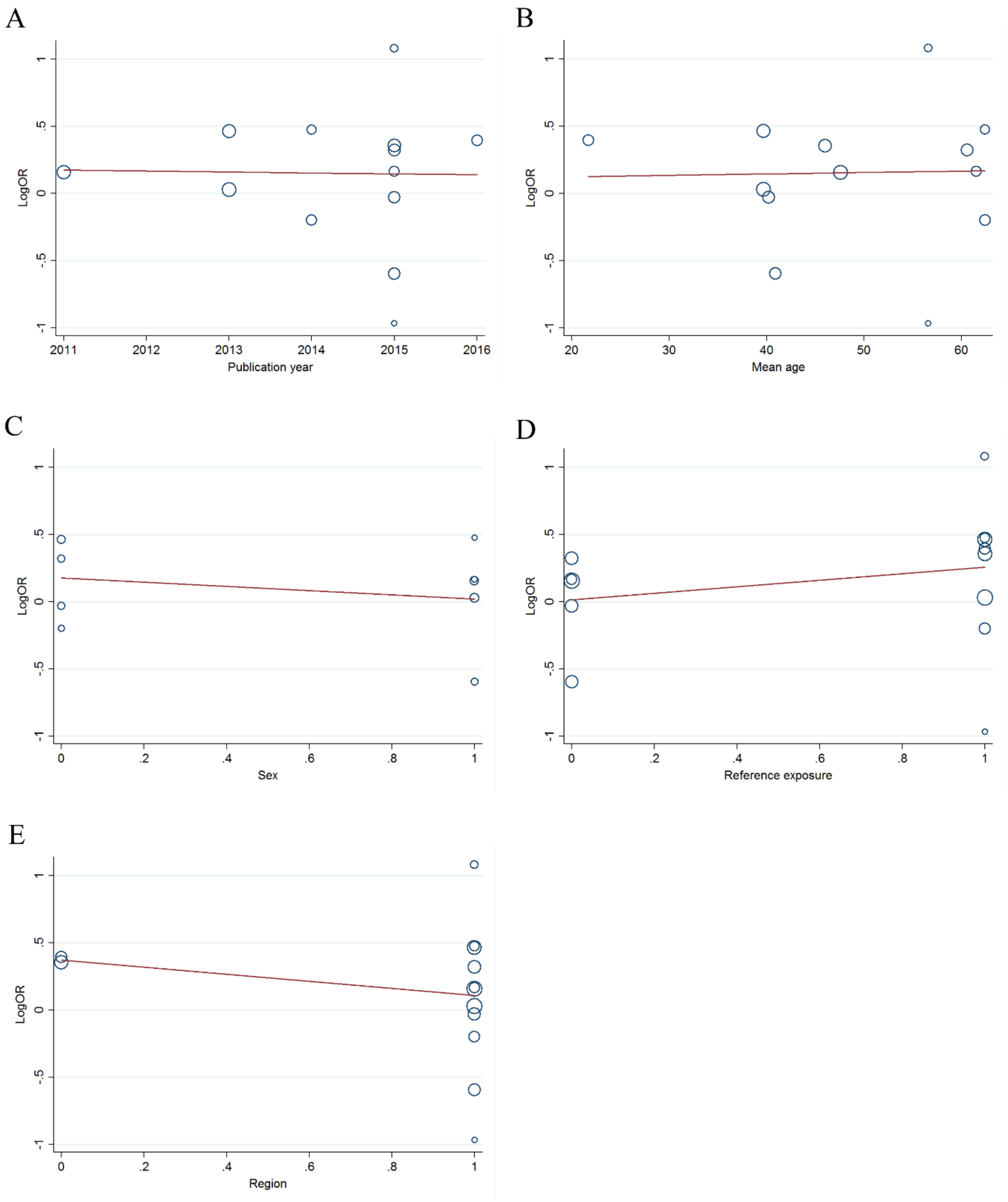

Supplement: Supplementary Information [file srep31956-s1.pdf]
